# Agouti‐related peptide neuronal silencing overcomes delayed puberty in neonatally underfed male mice

**DOI:** 10.1111/jne.13190

**Published:** 2022-08-19

**Authors:** Caroline Decourt, George A. D. P. Connolly, Caroline Ancel, Megan A. Inglis, Greg M. Anderson

**Affiliations:** ^1^ Centre for Neuroendocrinology and Department of Anatomy University of Otago School of Biomedical Sciences Dunedin New Zealand

**Keywords:** agouti‐related peptide, DREADD, mice, neonatally underfed, puberty onset

## Abstract

Agouti‐related peptide (AgRP) neurons are thought to indirectly regulate the activity of hypothalamic gonadotrophin‐releasing hormone neurons which control fertility. AgRP neurons also drive caloric intake and are modulated by metabolically‐relevant hormones, providing a link to the hypothalamic–pituitary‐gonadal axis. In mice expressing Cre‐dependant designer receptors (DREADDs) in AgRP neurons, we activated or silenced these neurons in vivo using the synthetic ligand clozapine‐N‐oxide (CNO) to observe the effect of AgRP neuron activity on timing of puberty. To validate these animals, we chronically treated both stimulatory (hM3Dq) and inhibitory (hM4Di) DREADD × AgRP‐Cre mice with CNO, observing a pronounced increase and decrease of food intake, respectively, consistent with the known orexigenic effects of these neurons. RNAscope was performed to visually confirm the activation of AgRP neurons. Puberty onset was assessed in males and females. There was no effect on preputial separation in males or vaginal opening and first oestrus in females after CNO treatment from day 26 to 30 to chronically modulate AgRP neurons. Next, to determine whether the delay in puberty onset occurring in response to neonatal underfeeding could be overcome by inhibiting AgRP neuronal activity, mice were raised in large (neonatally underfed) or normal litter sizes. The delay in puberty from underfeeding was completely reversed in CNO‐treated AgRP‐hM4Di male mice. These data highlight the inhibitory role of AgRP neurons to delay puberty onset when undernutrition occurs during the neonatal period, at least in male mice.

**Trail registration number:**

JNE‐22‐0081‐OA.R2

## Graphical abstract

Agouti‐related peptide (AgRP) neurons are thought to indirectly regulate the activity of hypothalamic gonadotrophin‐releasing hormone neurons which control fertility. AgRP neurons also drive caloric intake and are modulated by metabolically‐relevant hormones, providing a link to the hypothalamic‐pituitary‐gonadal axis. In mice expressing Cre‐dependant designer receptors (DREADDs) in AgRP neurons, we activated or silenced these neurons in vivo using the synthetic ligand CNO to observe the effect of AgRP neuron activity on timing of puberty. We showed that inhibition of AgRP neuronal activity (4Di) was able to overcome the adverse effects of nutritional restriction on puberty onset in males.

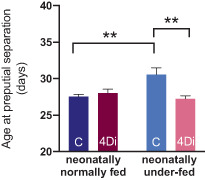

## 1 INTRODUCTION

Reproduction is an energy demanding function and thus, nutritional status and reproductive function are very closely associated. During times of low food abundance, animals must choose to allocate increased time to foraging at the expense of reproductive activity. Metabolic status and fertility are affected by many neuronal populations, one of which is the arcuate population of agouti‐related peptide (AgRP) neurons. These neurons drive food intake in response to situations of caloric need, and are modulated by metabolically‐relevant hormones such as leptin and insulin.^1^ They also provide a link to the hypothalamic–pituitary‐gonadal (HPG) axis, possibly through functional synaptic contact with GnRH neurons^2^, ^3^, ^4^ and/or with arcuate and anteroventral kisspeptin neurons.^5^, ^6^ It is likely that a complex network exists between AgRP neurons and other neurons of the GnRH neuronal network to coordinate reproductive activity.

AgRP neurons, located in the arcuate nucleus of the hypothalamus, coexpress the highly potent orexigenic peptides AgRP and neuropeptide Y (NPY), as well as gamma‐aminobutyric acid (GABA).^7^, ^8^, ^9^ AgRP is an inverse agonist of the melanocortin 3 and 4 receptors (MC3R and MC4R), inhibiting the anorexigenic effects of the melanocortin system neuropeptide α‐melanocyte‐stimulating hormone (MSH) and stimulating caloric intake.^7^, ^9^, ^10^ Intracerebroventricular administration of AgRP leads to an increase in bodyweight and food intake.^11^, ^12^

In leptin‐deficient, infertile male and female *ob/ob* mice, ablation of AgRP neurons lead to a restoration of fertility.^13^ Moreover, AgRP neuron‐specific leptin receptor knockout mice exhibited delayed onset of oestrous cyclicity, indicating that at least part of the actions of leptin occur directly on AgRP neurons. Remarkably, rescue of leptin receptors only in AgRP neurons is sufficient to rescue the disturbances seen in leptin receptor knockout mice.^14^ This provided evidence that the increased AgRP neuron signalling that results from the lack of inhibitory leptin actions leads to the suppression of the HPG axis, and that leptin signalling directly in AgRP neurons is sufficient and partially required for normal reproductive function.

Despite these findings regarding the role of leptin in AgRP neuronal actions, little is known about the role of AgRP neurons per se in reproductive function, particularly pubertal timing. In order to study this, we used chemogenetic designer receptors exclusively activated by designer drugs (DREADD) technology to inhibit or stimulate AgRP neuronal activity over relatively long time periods. After validating the AgRP‐Cre × Cre‐dependant DREADD mouse models, we examined the effect of AgRP neuronal modulation on puberty onset. We then attempted to overcome the delay in puberty onset that is known to occur in response to neonatal underfeeding^15^ by inhibiting AgRP neuronal activity.

## 2 MATERIALS AND METHODS

### 2.1 Mouse care

C57BL/6J mice were group housed (3–5 per cage) at a University of Otago animal facility under standard laboratory conditions (12 h on/off; lights on at 0630 h) and temperature‐controlled environment (22 ± 1°C). Experiments performed during the animal's dark phase were completed under red light to ensure that the circadian rhythm of these animals was not disturbed. Unless otherwise specified, food (standard rodent chow) and water were provided ad libitum. All experimental protocols were approved by the University of Otago Animal Ethics Committee.

### 2.2 Mouse model

DREADDs are mutated muscarinic acetylcholine G protein‐coupled receptors (GPCR) that are reported to respond specifically to nanomolar concentrations of clozapine‐N‐oxide (CNO), an otherwise pharmacologically inactive compound. When expressed only in specific cell types, this chemogenetic technology enables precise activation and inhibition of these neuronal populations noninvasively in vivo.

We utilized two different types of Cre‐dependant DREADD mice. The first of these was the stimulating hM3Dq‐flox mouse line (Jackson Laboratories, # 026220) that enables control of Gq‐mediated signalling, and the second was the inhibiting hM4Di‐flox mouse line (Jackson Laboratories, # 026219) that enables control of Gi‐mediated signalling.^16^ LoxP sites flank a stop codon upstream to the hM3Dq and hM4Di gene sequences. To specifically target the AgRP population, both mouse strains were individually crossed with an AgRP‐Cre mouse (Jackson Laboratories, # 012899), where Cre recombinase is exclusively expressed in cells encoding the AgRP gene.^17^ When crossed, the sequence between the LoxP sites (i.e., the stop‐codon) is excised by Cre‐recombinase, allowing the hM3Dq or hM4Di receptors to be expressed exclusively in AgRP‐expressing cells.

We generated male and female mice expressing AgRP‐specific excitatory DREADDs (AgRP‐Cre × hM3Dq‐flox; AgRP‐hM3Dq) and inhibitory DREADDs (AgRP‐Cre × hM4Di‐flox; AgRP‐hM4Di), as well as control mice (AgRP‐Cre siblings not inheriting the DREADD genes). Control mice received the same CNO treatment in each experimental protocol as the AgRP‐hM3Dq or AgRP‐hM4Di mice in order to control for any possible off‐target effects of CNO.

To identify the genotype of the mice, a polymerase chain reaction (PCR) analysis of genomic DNA was used.^14^, ^16^ The AgRP‐Cre line was genotyped using AgRP‐Cre primers (common‐forward: 5′‐GCT TCT TCA ATG CCT TTT GC‐3′; wt‐reverse: 5′‐GTG TGT GGT TCC AGC ATG AC‐3′; mutant‐reverse 5′‐GG AAC TGC TTC CTT CAC GA‐3′; annealing temperature 60°C; product size indicating the Cre allele: 280 bp). Floxed DREADD mice were identified using the following PCR primer sets and annealing temperatures: 5′‐AAG GGA GCT GCA GTG GAG TA‐3′ (wild‐type forward primer), 5′‐ CCG AAA ATC TGT GGG AAG TC‐3′ (wild‐type reverse primer), 5′‐ ATG TCT GGA TCC CCA TCA AG‐3′ (mutant forward primer for both hM3Dq and hM4Di), 5′‐GAT GTT GCC GAT GAT GGT CAC‐3′ (mutant hM3Dq reverse primer), and 5′‐ GAA GGC GCC TAT GAT GAG ATC‐3′ (mutant hM4Di reverse primer); annealing temperature 55°C; product size indicating the floxed and wild‐type alleles: 440 and 300 bp, respectively.

### 2.3 Treatments

For chronic manipulation of AgRP neurons, CNO was dissolved in 0.2% DMSO/water at 0.01 mg/ml concentration. The mice were administered CNO (approximatively 5 mg/kg/day) through their drinking water, assuming that a mouse drinks 5–10 ml per day.^18^ Water was changed daily to ensure that the drug was fresh, and the liquid was protected from the light.

For acute manipulation of AgRP neurons, CNO was dissolved in 0.5% DMSO/saline at 0.1 mg/ml concentration. Mice were administered CNO (1 mg/kg) via a subcutaneous injection.

### 2.4 Bodyweight and food intake measurements

AgRP neurons are orexigenic, therefore, to validate both the AgRP‐hM3Dq and AgRP‐hM4Di mice, as well as the dose of CNO used, bodyweight was measured in the presence of chronic CNO administration and food intake was measured in the presence of acute CNO administration. A significant increase in these measures in the AgRP‐hM3Dq mice and a significant decrease in AgRP‐hM4Di mice would confirm AgRP neuronal activation and inhibition, respectively, indicating that the activity of these neurons was indeed being altered as expected in response to the dose of CNO used.^6^, ^19^, ^20^ To this end, AgRP‐hM3Dq (*n* = 5), AgRP‐hM4Di (*n* = 14), and control male mice (*n* = 9), aged postnatal day (PND) 50, were chronically administered CNO (~5 mg/kg/day) through their drinking water. Bodyweight was recorded every 24 h at 0900 h for 4 days. To measure food intake, the same male mice were individually caged and acutely administered CNO (1 mg/kg) via a subcutaneous injection at PND 70. AgRP‐hM3Dq (*n* = 3) and control mice (*n* = 3) were injected at the beginning of their light phase (0630 h), the 12‐h period where they consume the least quantity of food.^21^ Food was removed from the cage for 30 min, and a single preweighed pellet was then placed in the cage and weighed at +15, 30, 60, 180 and 330 min. AgRP‐hM4Di (*n* = 3) and control mice (*n* = 3) were injected at the beginning of the dark phase (1830 h), the 12‐h period where they consume the greatest quantity of food.^21^ Food was removed from the cage for 30 min, and a single preweighed pellet was then placed in the cage and weighed at +60, 90, 180, 240 and 360 min).

### 2.5 Glucose and insulin tolerance tests

To assess glucose homeostasis, a glucose tolerance test (GTT) was performed in adult AgRP‐hM3Dq (*n* = 7), AgRP‐hM4Di (*n* = 6) and control (*n* = 9) male mice. Mice were fasted for 8 h and received an acute subcutaneous CNO injection (1 mg/kg) 30 min before the GTT test. Blood glucose levels were measured in tail vein blood using a glucose meter (CareSensN, model GM505PAD) at baseline, 15, 30, 60, and 90 min after intraperitoneal challenge of glucose (1 g/kg).

To assess insulin resistance, an insulin tolerance test (ITT) was performed in AgRP‐hM3Dq (*n* = 7), AgRP‐hM4Di (*n* = 6) and control (*n* = 9) male mice. Mice were fasted for 4 h and received an acute subcutaneous CNO injection (1 mg/kg) 60 min before the ITT test. Blood glucose levels were measured as described above at baseline, 15, 30, 60, 90 and 120 min after intraperitoneal challenge of insulin (0.25 U/kg; Sigma‐Aldrich; # 15523; insulin from porcine pancreas).

### 2.6 Immunohistochemistry

Nonfasted male mice AgRP‐hM3Dq (*n* = 3) and AgRP‐hM4Di (*n* = 3) were deeply anaesthetized using an intraperitoneal injection of sodium pentobarbital (240 mg/kg) and perfused transcardially with 4% paraformaldehyde (PFA) in 0.1 M phosphate buffer (PB). The brains were promptly removed from the skulls and postfixed with 4% PFA at 4°C overnight, and retained in 30% sucrose in 0.1 M PB at 4°C until they sank, followed by sectioning using a microtome with a freezing stage into three identical sets of 30 μm thick coronal free‐floating sections.

Briefly, sections were washed three times for 10 min each in 0.05 M TBS‐TX and incubated in 1% hydrogen peroxide for 30 min at room temperature. Sections were incubated overnight in primary antibody for HA‐tag (1:2000; monoclonal rabbit anti‐hemagglutinin [HA] HA‐Tag, Cell Signalling Technology, Cat#3724, RRID:AB_1549585) in a blocking solution of 2% normal goat serum in TBS‐TX‐BSA at 4°C. On the subsequent day, sections were washed three times for 10 min each and incubated in secondary antibody, a biotinylated goat anti‐rabbit (1:1000; Vector laboratories, Cat#BA1000, RRID:AB_2313606) for a period of 1 h at room temperature. Sections were washed three times for 10 min each and incubated in avidin‐biotin‐peroxidase solution (Elite ABC Kit, Vector laboratories, Cat# PK6100, RRID:AB_2336819) for a period of 1 h at room temperature. Sections were washed three times for 10 min each before being incubated in DAB solution (SigmaFast DAB with metal enhancer, Sigma‐Aldrich, #D0426) for approximately 3 min. Then, the DAB reaction was stopped and sections were mounted, dehydrated and coverslipped with glass microscope slides using Depex.

### 2.7 RNAscope

*c‐Fos* was used in this experiment as a marker of neuronal activation, to compare the effects of CNO on AgRP activity between the three experimental groups.

CNO was administered acutely via subcutaneous injection (1 mg/kg) at 1000 h, 90 min prior to sacrifice, on nonfasted adult male mice. Brains from AgRP‐hM3Dq, AgRP‐hM4Di and control mice (*n* = 2 per group) were collected and immediately frozen. Brains were embedded in optimal cutting temperature (OCT) compound, then frozen sections were cut into three identical sets of 16 μM coronal sections containing the arcuate nucleus using a cryostat (Leica CM1950), and mounted onto Superfrost slides and stored at **−**80°C. Commercially available RNA probes for *Agrp* (ADV400711) and *Cfos* (ADV316921C2) and a duplex mouse reagent kit (ADV322436) were purchased from Advanced Cell Diagnostics USA and used according to the manufacturer's instructions. Sections between −1.5 to −2 mm relative to bregma were counted.

### 2.8 Experiment 1: Effect of chronic AgRP stimulation and inhibition on puberty onset

AgRP‐hM3Dq (*n* = 7), AgRP‐hM4Di (*n* = 6), and control (*n* = 9) male mice, and AgRP‐hM3Dq (*n* = 6), AgRP‐hM4Di (*n* = 7), and control (*n* = 10) female mice, from litter size ranges 4–10, were chronically treated with CNO (~5 mg/kg/day) via their drinking water from PND 26 to 30. Timing of puberty was determined using daily visual assessment of preputial separation and vaginal opening as the anatomical indications of puberty onset in male and female mice, respectively. Once vaginal opening had occurred, the age at first oestrus was determined by vaginal cytology analysis as an additional marker of puberty in females.

### 2.9 Experiment 2: Effect of chronic AgRP neuron inhibition on puberty onset in neonatally underfed and neonatally normally fed pups

We were interested in determining whether chronic AgRP inhibition was sufficient to counteract the delay in puberty onset observed under neonatal underfeeding conditions.^15^ To test this, AgRP‐Cre mice were crossed with Cre‐dependant hM4Di‐flox mice and litter sizes were manipulated on PND 2 by randomly distributing pups among mothers such that large litters (neonatally underfed) had 12 pups and normal litters (neonatally normally fed) had six pups. All mice were weaned at PND 21 and given ad libitum access to food and water.

Female pups from large litters were used if their bodyweight was under 12 g at PND 25, and females from normal litters were used if their bodyweight was over 12 g at this age. For males, the inclusion criteria was below or above 13 g for large and normal litters, respectively. No more than two pups per group were excluded by these criteria.

Female and male pups were chronically treated with CNO (~5 mg/kg/day) via their drinking water from PND 26 to 30 (*n* = 8 per group). Bodyweight was recorded at 0900 h daily from PND 25 to 31, and then on PND 35. The age of puberty onset was visually assessed daily from PND 25 by vaginal opening, first oestrus and preputial separation measurement.

### 2.10 Statistical analysis

Statistical analyses were performed using commercial software (GraphPad Prism; GraphPad Software Inc). For all results, the significance threshold was placed at *α* = 0.05.

**FIGURE 1:**
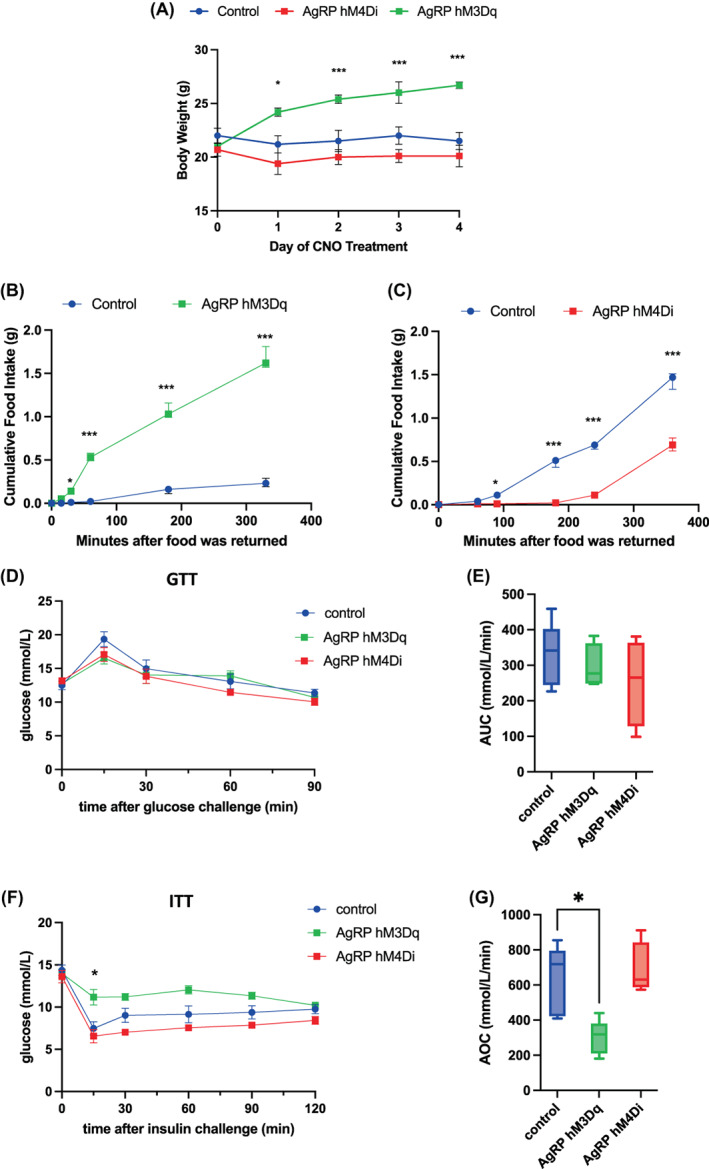
Validation of AgRP neuronal silencing and activation mouse models. (A) Effect of chronic clozapine‐N‐oxide (CNO) administration (~5 mg/kg/day in drinking water) on bodyweight in AgRP‐hM3Dq (*n* = 5), AgRP‐hM4Di (*n* = 14), and control (*n* = 9) male mice, over 4 days of chronic CNO administration. (B) Effect of acute CNO administration (1 mg/kg sc) on food intake in AgRP‐hM3Dq and control mice (*n* = 3/group). CNO was administered at the beginning of the light period. (C) Effect of acute CNO administration (1 mg/kg sc) on food intake in AgRP‐hM4Di and control mice (*n* = 3/group). CNO was administered at the beginning of the dark period. (D) Glucose tolerance test (GTT) in AgRP‐hM3Dq (*n* = 7), AgRP‐hM4Di (*n* = 6), and control (*n* = 9) male mice, after 8 h fasting, with acute CNO administration (1 mg/kg sc) 30 min before glucose challenge (1 g/kg ip). (E) Analysis of GTTs with areas under the curves (AUC). (F) Insulin tolerance test (ITT) in AgRP‐hM3Dq (*n* = 7), AgRP‐hM4Di (*n* = 6), and control (*n* = 9) male mice, after 4 h fasting, with acute CNO administration (1 mg/kg sc) 60 min before insulin challenge (0.25 U/kg ip). The asterisk shows a significantly smaller glucose reduction in AgRP‐hM3Dq compared to control mice at +15 min. (G) Analysis of ITTs with areas over the curves (AOC). (A–D and F) Values are presented as mean ± SEM. Statistical comparisons were made using a two‐way ANOVA followed by a Holm‐Šídák post‐hoc test. (E and G) Values are presented as median and interquartile range. Statistical comparisons were made using a Kruskal‐Wallis test, followed by a Dunn's multiple comparison test. *p < .05, ***p < .001

Two‐way ANOVA with repeated measures with time and genotype as the factors, followed by the Holm‐Šídák multiple comparison post‐hoc test if a statistically significant result was found, was applied to bodyweight, food intake, GTT and ITT experimental data from Figure 1A–D,F.

In experiment 2, a three‐way ANOVA was used for repeated measures followed by the Tukey's multiple comparison test (applied to bodyweight data in Figures 4A and 5A, where time, genotype and neonatal nutritional condition were the factors). Then, to analyse the interaction between the genotype and the neonatal nutritional condition, a two‐way ANOVA was used, followed by the Holm‐Šídák multiple comparison post‐hoc test (applied to data from Figure 4B–D and 5B–E).

Nonparametric data (RNAscope experiment, age at preputial separation, at vaginal opening and at first oestrus, area under the curves for GTT, and area over the curves for ITT) was analysed using Kruskal‐Wallis test followed by the Dunn's multiple comparison post‐hoc test. The assumptions to perform a Kruskal–Wallis test were that the data distribution did not fit a Gaussian distribution or have equal variances, or the data were essentially ordinal (i.e., puberty age).

## 3 RESULTS

### 3.1 Validation of the mouse models

#### 3.1.1 Effect of chronic AgRP neuron stimulation and inhibition on bodyweight

There was a significant treatment group effect on bodyweight (*F*_[2125]_ = 28.70, *p* < .001). A significant increase was observed in bodyweight of CNO‐treated AgRP‐hM3Dq mice, at days 1 (*p* < .05), 2, 3 and 4 (*p* < .001) of CNO treatment, compared to the controls, while no significant decrease was observed in the bodyweight of CNO‐treated AgRP‐hM4Di animals compared to controls during CNO treatment (Figure 1A).

#### 3.1.2 Effect of acute AgRP neuron stimulation and inhibition on food intake

Food intake was recorded after CNO was acutely administered via subcutaneous injection in AgRP‐hM3Dq (Figure 1B) and AgRP‐hM4Di (Figure 1C) versus control mice. For the AgRP‐hM3Dq animals, a significantly greater cumulative food intake was observed compared to control mice at 30 (*p* < .05), 60 (*p* < .001), 180 (*p* < .001) and 320 (*p* < .001) min after food was returned (Figure 1B). For the AgRP‐hM4Di animals, a significant decrease in cumulative food intake was observed compared to control mice at 90 (*p* < .05), 180 (*p* < .001), 300 (*p* < .001), and 340 (*p* < .001) min after food was returned (Figure 1C).

#### 3.1.3 Effect of acute AgRP neuron stimulation and inhibition on glucose levels

To investigate the effect of AgRP modulation on glucose levels, GTT and ITT were performed in AgRP‐hM3Dq, AgRP‐hM4Di and control mice after CNO administration. There was no significant difference in the increase of glucose levels after glucose challenge between control, AgRP‐hM3Dq and AgRP‐hM4Di mice (Figure 1D). AgRP neuronal modulation did not affect overall blood glucose responses during the GTT as assessed by the areas under the curves, which were not different between groups (Figure 1E).

A significant decrease of glucose was observed in AgRP‐hM4Di and control, but not AgRP‐hM3Dq mice 15 min after insulin challenge (*p* < .05 for AgRP‐hM3Dq compared to control mice concentrations at 15 min) (Figure 1F). The areas over the curves were significantly lower in AgRP‐hM3Dq mice compared to control mice (*p* < .05), but not significantly higher in AgRP‐hM4Di mice compared to control mice (Figure 1G).

Blood glucose levels were unaffected by CNO administration in the sample collected prior to glucose or insulin treatments (data not shown).

### 3.2 Immunohistochemistry and RNAscope

**FIGURE 2:**
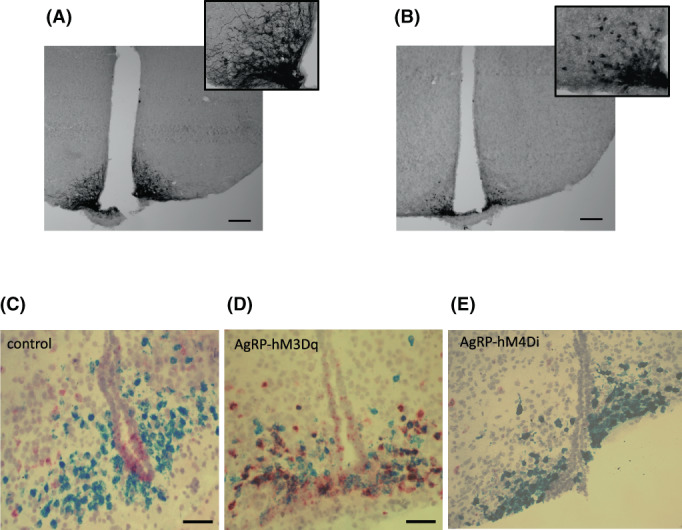
Validation of hM3Dq and hM4Di expression by immunohistochemistry and AgRP neuronal silencing and activation by RNAscope. Representative examples of HA‐tag immunoreactivity in the ARC of AgRP‐hM3Dq (A) and AgRP‐hM4Di (B) mice. Effect of acute clozapine‐N‐oxide (CNO) administration (1 mg/kg sc) on AgRP neuronal activity, using RNAscope probe targeting *Agrp* (blue) and *cFos* (red) on arcuate nucleus in control (C), AgRP‐hM3Dq (D) and AgRP‐hM4Di (E) male mice. (F) Percentage of colocalization of *Agrp* with *cFos* in AgRP‐hM3Dq, AgRP‐hM4Di and control mice. (G) Average of AgRP neurons per section in AgRP‐hM3Dq, AgRP‐hM4Di and control mice. *N* = 2 per group. Values are presented as median and interquartile range. Statistical comparisons were made using a Kruskal‐Wallis test, followed by a Dunn's multiple comparison test. *p < .05. Scale bar represents 100 μm

The Cre‐dependant DREADDs hM3Dq and hM4Di have an HA tag that gets turned on when the DREADD is expressed. Immunohistochemistry for HA was used as a marker of hM3Dq and hM4Di expression. The localisation of the staining was concentrated in the ventrolateral ARC in AgRP‐hM3Dq (Figure 2A) and AgRP‐hM4Di (Figure 2B) mice, where AgRP neurons are located.

*Agrp* and *c‐Fos* mRNA expression were quantified in the arcuate nucleus (3 sections per animal) to determine the percentage of AgRP neuronal activation after CNO acute administration. After CNO administration, the percentage of *c‐Fos* and *Agrp* colocalisation was significantly higher in AgRP‐hM3Dq mice compared to controls (67.7 ± 2.9% vs. 43.3 ± 6.8%, respectively; *p* < .05) (Figure 2C,D,F), and not significantly lower in AgRP‐hM4Di mice compared to controls (39.3 ± 1.4% vs. 43.3 ± 6.8%, respectively; *p* = .95) (Figure 2C,E,F). The average of AgRP cell number per section was not significantly different between the three groups (Figure 2G). Very little *c‐Fos* expression was observed in non‐AgRP neurons in AgRP‐hM3Dq and AgRP‐hM4Di mice, similar to what was observed in control mice, confirming the specificity of DREADD expression to these cells.

### 3.3 Experiment 1: Effect of chronic AgRP neuron stimulation and inhibition on puberty onset

**FIGURE 3:**
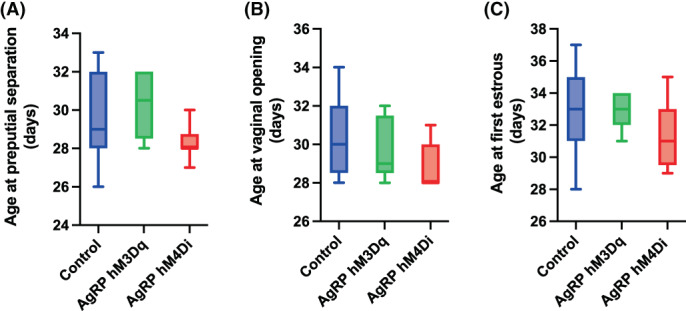
Puberty onset in AgRP‐hM3Dq, AgRP‐hM4Di and control mice chronically treated with clozapine‐N‐oxide (CNO) from postnatal day 26 to 30. (A) Preputial separation was used as an anatomical indicator of puberty onset in male mice. (B) Vaginal opening and (C) first estrus were used as an anatomical indicator of puberty onset in female mice. Values are presented as median and interquartile range. Statistical comparisons were made using a Kruskal‐Wallis test, followed by a Dunn's multiple comparison test. *p < .05. Control male: *n* = 9; Inhibited male: *n* = 6; Stimulated male: *n* = 7. Control female: *n* = 10; Inhibited female: *n* = 7; Stimulated female: *n* = 6

CNO was chronically administered from PND 26 to 30. The age at preputial separation and at vaginal opening and first oestrus were used as an anatomical indicator of puberty onset in male mice (Figure 3A) and female mice (Figure 3B,C), respectively.

No significant difference was observed in the age at preputial separation in CNO‐treated AgRP‐hM3Dq and AgRP‐hM4Di mice compared to controls, and no significant differences were observed in the age at vaginal opening and at first oestrus in CNO‐treated AgRP‐hM3Dq and AgRP‐hM4Di mice compared to controls.

### 3.4 Experiment 2: Effect of chronic AgRP inhibition on puberty onset in neonatally underfed pups compared to neonatally normally fed pups

We next wanted to determine whether the delay in puberty onset observed in neonatally underfed mice^15^ could be overcome by AgRP neuron inhibition.

#### 3.4.1 Males

**FIGURE 4:**
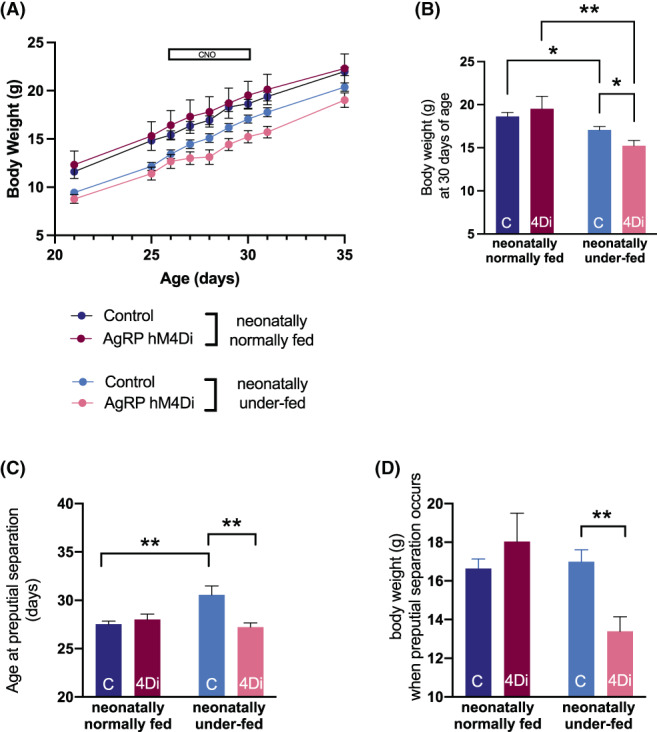
Chronic AgRP inhibition overcomes the effect of neonatal underfeeding on puberty onset in male mice. (A) Bodyweight in AgRP‐hM4Di and control male mice, neonatally normally fed and neonatally underfed from day 21 to 35. The horizontal bar represents the period of chronic clozapine‐N‐oxide (CNO) administration (5 mg/kg/day). (B) Bodyweight in AgRP‐hM4Di and control male mice, neonatally normally fed and neonatally underfed at the end of CNO treatment (day 30). (C) Age at preputial separation in AgRP‐hM4Di and control neonatally normally fed and neonatally underfed mice. (D) Bodyweight at the age when preputial separation occurred. Values are presented as mean ± SEM. (A) Statistical comparisons were made using a three‐way ANOVA followed by a Tukey's multiple comparison post‐hoc test. (B–D) Statistical comparisons were made using a two‐way ANOVA followed by a Holm‐Šídák post‐hoc test. *p < .05, **p < .01. *N* = 8 per group

There were a significant age effect (*F*_(3.053,78.94)_ = 1124, *p* < .0001) and neonatal feeding condition effect (*F*_(1,27)_= 42.33, *p* < .0001) on bodyweight (Figure 4A). Before CNO administration (PND 26), the bodyweight of neonatally underfed pups in the control and AgRP‐hM4Di groups was reduced by 30.8 and 28.4%, respectively, compared to the corresponding neonatally normally fed pups (*p* < .01). Throughout the period of chronic CNO administration (PND 26–30), this difference was maintained in the controls and became exacerbated in AgRP‐hM4Di animals, despite the fact all mice had ad libitum access to food post‐weaning. After completion of chronic CNO administration (PND 30), the bodyweight of neonatally underfed pups in the control and AgRP‐hM4Di groups was still significantly reduced compared to compared to the corresponding neonatally normally fed pups (*p* < .05 and *p* < .01, respectively) (Figure 4B). Five days after completion of CNO administration (PND 35), the effect of neonatal underfeeding was still significant.

There was a significant genotype effect on age at preputial separation (*F*_(1,28)_= 5.57, *p* < .05). The age at preputial separation was delayed by 3 days in neonatally underfed control mice compared to neonatally normally fed control mice (*p* < .01) (Figure 4C). This effect was completely reversed in neonatally underfed AgRP‐hM4Di mice compared to neonatally underfed controls, with a similar age at preputial separation observed in neonatally underfed AgRP‐hM4Di and both neonatally normally fed groups, despite the markedly compromised bodyweight caused by the additive effects of neonatal underfeeding and AgRP neuronal silencing (Figure 4D). The bodyweight at which preputial separation was observed was significantly lower in CNO‐treated neonatally underfed AgRP‐hM4Di mice compared to neonatally underfed control mice (Figure 4D).

#### 3.4.2 Females

**FIGURE 5:**
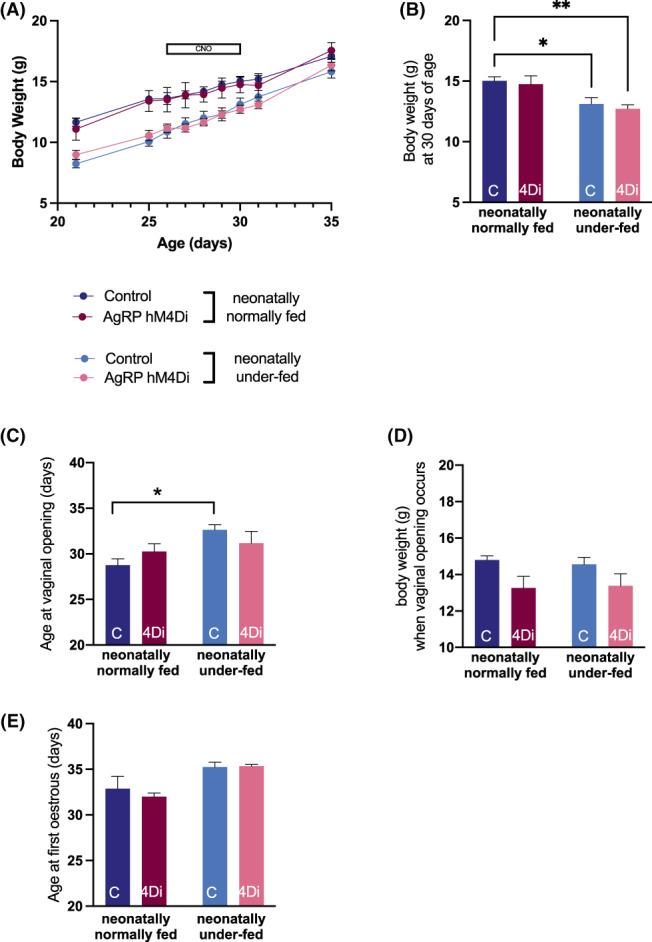
Chronic AgRP inhibition does not overcome the effect of neonatal underfeeding on puberty onset in female mice. (A) Bodyweight in AgRP‐hM4Di and control female mice, neonatally normally fed and neonatally underfed from day 21 to 35. The horizontal bar represents the period of chronic clozapine‐N‐oxide (CNO) administration (5 mg/kg/day). (B) Bodyweight in AgRP‐hM4Di and control female mice, neonatally normally fed and neonatally underfed at the end of CNO treatment (day 30). (C) Age at vaginal opening in AgRP‐hM4Di and control, neonatally normally fed and neonatally underfed mice. (D) Bodyweight at the age when vaginal opening occurred. (E) Age at first estrus in AgRP‐hM4Di and control, neonatally normally fed and neonatally underfed mice. Values are presented as mean ± SEM. (A) Statistical comparisons were made using a three‐way ANOVA followed by a Tukey's multiple comparison post‐hoc test. (B–D) Statistical comparisons were made using a two‐way ANOVA followed by a Holm‐Šídák post‐hoc test. *p < .05, ** *p* < .01. *N* = 8 per group

There were a significant age effect (*F*_(3.643,80.14)_= 301.3, *p* < .0001) and neonatal feeding condition effect (*F*_(1,22)_ = 2352, *p* < .0001) on bodyweight (Figure 5A). Before CNO administration (PND 26), the bodyweight of neonatally underfed control and AgRP‐hM4Di pups was significantly reduced by 20.6% and 17.8%, respectively compared to neonatally normally fed control and AgRP‐hM4Di pups (*p* < .01). Throughout the period of chronic CNO administration (PND 26–30), this difference was maintained in the control and AgRP‐hM4Di mice. After completion of chronic CNO administration (PND 30), the bodyweight of neonatally underfed pups in the control groups was still significantly reduced compared to compared to the corresponding neonatally normally fed pups (*p* < .05). However, no significant difference was observed in the AgRP‐hM4Di group (Figure 5B). Five days after completion of CNO administration (PND 35), the bodyweight of neonatally underfed control and AgRP‐hM4Di mice was not significantly different from neonatally normally fed control and AgRP‐hM4Di mice.

There was a significant treatment group effect on age at vaginal opening (*F*_(1,22)_ = 7.22, *p* < .05). Similarly to what has been shown in previous experiments in female mice,^15^ the age at vaginal opening was delayed in neonatally underfed versus normally fed control mice (32.6 ± 0.6 days vs. 28.7 ± 0.7 days, respectively) (*p* < .05). While this effect was not completely reversed in neonatally underfed AgRP‐hM4Di mice compared to neonatally underfed controls (*p* > .99), the age at vaginal opening was nevertheless not significantly different between neonatally underfed AgRP‐hM4Di mice and both neonatally normally fed groups, despite the markedly compromised bodyweight caused by neonatal underfeeding (Figure 5C). The bodyweight at which vaginal opening was observed was not significantly different between groups (Figure 5D). Similarly to what has been observed for the age at vaginal opening, there was a significant treatment group effect on age at first oestrus (*F*_(1,22)_ = 8.83, *p* < .01). However, the age at first oestrus was not significantly different between groups (Figure 5E).

## 4 DISCUSSION

The neuropeptides secreted by AgRP/NPY neurons are well‐known to be highly orexigenic^8^ and critically involved in the drive to forage for food at the expense of other activities.^22^ Their involvement in reproductive processes is less well understood. AgRP neurons have been shown to make contact with GnRH neurons in lactating rats.^2^ AgRP (as well as NPY) can influence GnRH neuronal activity. Roa et al. reported primarily inhibitory, but also some excitatory effects of these two neuropeptides.^4^ Recently, viral addition of activating (hM3Dq) DREADDs into AgRP neurons was used to demonstrate the ability of these neurons to impair estrous cycles and also the time taken to conceive after mating in female mice.^6^ Based on brain slice recordings from GnRH and kisspeptin neurons, the authors suggested that it is the latter cell type that is the direct target of AgRP neurons. The present study is the first to use Cre‐dependant silencing DREADDs to investigate the effects of manipulating AgRP neuronal activity on puberty onset. Our results show that inhibition of AgRP neuronal activity can overcome the adverse effects of nutritional restriction on puberty onset in males.

AgRP is well known to have a strong orexigenic effects, driving food intake behaviour. Chronic CNO administration for 4 days led to a significant increase in bodyweight in AgRP‐stimulated adult mice, in accordance with previous observations.^6^, ^19^, ^20^ A dramatic increase in cumulative food intake was observed in AgRP‐hM3Dq mice compared to control adult mice, from 30 min after acute CNO administration, performed at the beginning of the light phase when the food intake is the least.^21^ The increased orexigenic behaviour of these animals is in accordance with the significant increase in food intake observed after an intracerebroventricular injection of AgRP,^12^ and with previous evaluation of CNO treatment on AgRP neuron‐stimulated animals.^6^, ^20^

Conversely, no significant decrease in bodyweight was observed in mice that were AgRP‐silenced, during CNO treatment, compared to control adult mice. The absence of bodyweight suppression in response to AgRP neuronal inhibition is consistent with the minor effects of deletion of *Agrp* and/or *Npy* genes in mice^23^, ^24^, ^25^ and of AgRP neuronal ablation, at least in young mice.^26^, ^27^ This suggests that compensatory mechanisms are able to offset the lack of orexigenic influence from AgRP and NPY peptides, or that in nonfasted situations their basal activity is low anyway. A significant decrease in cumulative food intake was observed in AgRP‐hM4Di compared to control adult mice, from 90 min after acute CNO administration at the beginning of the dark phase, when the food intake is the greatest.^21^ This much more pronounced effect was not unexpected, considering the upregulation and strong orexigenic effect of AgRP at this time of day.^28^

Blood glucose levels were not affected after inhibition or stimulation of AgRP neuronal activity, however a change in insulin sensitivity was observed after acute modulation of AgRP neurons. Stimulation of AgRP neurons causes insulin resistance. These data are in accordance with previous reports showing that DREAAD‐mediated activation of AgRP neurons had no effect on glucose tolerance but decreased insulin sensitivity in mice,^29^, ^30^ although others showed a significant effect of chemogenetic AgRP neuronal activation and no effect of optogenetic activation on glucose tolerance.^29^ The latter chemogenetic effect may reflect the fact that those authors used double the glucose dose and fasting period than we did in the current experiment.

*c‐Fos* was used to quantify the change of neuronal activity between the three experimental groups. An increase was observed in AgRP‐hM3Dq mice after CNO injection; however, a significant decrease of activity in AgRP‐hM4Di was not observed. This may reflect an already low activity of AgRP neurons in the nonfasted state, or perhaps the limitations of using *c‐Fos* coexpression to accurately quantify neuronal activity in the unstimulated state. In addition, it is possible that the inhibitory DREADDs only dampened AgRP neural activity rather than completely eliminating it.^31^

Preputial separation, vaginal opening and first oestrus were used as an anatomical marker of puberty occurrence. Somewhat surprisingly, we observed no delay of puberty onset in AgRP neuron‐stimulated animals in both sexes, given the previous evidence for inhibitory actions of these neurons.^6^ This may have been due to the increased bodyweight in AgRP‐stimulated mice that could offset the inhibitory effects of AgRP and NPY neuropeptides on GnRH and/or kisspeptin neurons, as prepubertal obesity often advances puberty onset.^32^ The expected advancement of puberty onset in AgRP neuron‐inhibited animals was also not observed in both sexes. While it has been previously shown that induction of AgRP deficiency in leptin receptor‐deficient females does restore normal puberty timing,^5^ in the nonfasting and leptin signalling‐intact state, activity of AgRP neurons is already low and inhibiting these neurons further probably did not lead to a marked change in AgRP levels. Alternatively, a longer period of stimulation/inhibition of AgRP neurons may be required to affect puberty onset under normal feeding conditions. Our rationale for treating with CNO from PND 26 rather than an earlier age was to avoid adaptation or compensatory mechanisms, as well as to lessen the likelihood of obtaining an effect on puberty that was secondary to altered bodyweight caused by an extended period of treatment.

Considering the increase of *Agrp* mRNA expression during undernutrition,^8^ we decided to inhibit AgRP neurons to attempt to rescue the delay in puberty onset observed during underfeeding conditions.^15^ We used cross‐fostering to alter the nutritional status of pups. This led to the expected differences in bodyweight between the neonatally underfed and neonatally normally fed mice in both sexes. Before CNO treatment, at day 26, neonatally underfed mice exhibited a ~20%–30% lower bodyweight compared to neonatally normally fed mice, in both sexes. AgRP neuronal silencing from day 26 to 30 exacerbated this effect of neonatally underfeeding male mice, but had no effect on bodyweight of neonatally normally‐fed mice. This is consistent with the idea that the effect of AgRP silencing is most evident when the activity of these neurons is naturally high (i.e., in underfed animals). By contrast, the inability of AgRP silencing to cause a bodyweight reduction in neonatally normally‐fed male mice that were approaching their adult body size (see Figure 4), may simply reflect the greater drive to eat of growing animals. No catch‐up growth occurred during the D26–D35 period in male mice that were neonatally underfed, which is consistent with other studies.^33^ However, female mice that were neonatally underfed exhibited catch‐up growth during the D26–D35 period. This sexual dimorphism in catch‐up growth capacity has already been shown in rats.^34^, ^35^

As expected, the age at preputial separation and at vaginal opening was delayed in neonatally underfed control mice compared to neonatally normally fed control mice. This is consistent to what has been shown in previous experiments in female mice.^15^ However, in both sexes, their reduced growth rate meant that their bodyweight was similar to normally‐fed mice at the time of puberty onset. Such findings have led to the idea that a threshold bodyweight, or more likely adiposity level, is required to puberty onset, although evidence from overfed animals suggests that bodyweight and composition are not driving factors for puberty onset in male mice.^36^ Remarkably, AgRP neuronal silencing was sufficient to completely restore the delay of puberty onset caused by underfeeding observed in control males. This finding is reminiscent of the effects of AgRP deficiency in leptin receptor‐deficient mice on puberty timing.^5^ Interestingly, the combined effects of growth retardation and advancement of pubertal age meant that the bodyweight at the time when preputial separation occurred was very low in AgRP neuron‐inhibited neonatally underfed mice compared to other groups. The occurrence of preputial separation at such a low bodyweight in the present study provides evidence of the magnitude of HPG axis restraint normally exerted by AgRP neurons during the prepubertal period, especially in underfed animals.

In this study, AgRP neuronal silencing consistently overcame the puberty‐delaying effect of neonatal underfeeding in males, but not in females. Little is known about sex differences in how AgRP neurons modulate puberty timing. In both males and females, ablation of AgRP neurons restores fertility in leptin‐deficient mice^13^ and rescue of leptin receptors only in AgRP neurons enables puberty onset and fertility in leptin receptor knockout mice of both sexes.^14^ However, sexually dimorphic kisspeptin neurons, rather than GnRH neurons, may be the primary direct target of AgRP neurons,^6^ and recent experiments have demonstrated male‐specific susceptibility of puberty onset to metabolically‐relevant kisspeptin neuronal manipulation.^37^ On the other hand, the significant effects observed in males but not in females might simply be due to the timing of CNO administration relative to puberty timing. The PND 26–30 CNO treatment period may have been optimal for males but not for females with regard to the relative ages at which the HPG axis matures and become responsive to AgRP neuronal inputs.

The chemogenetically silenced AgRP neurons presumably attenuated their secretion of the neuropeptides AgRP and NPY, as well as the neurotransmitter GABA. It is not possible to determine from this study which of these neurochemicals is the primary effector for withholding puberty onset. Evidence from other studies shows that GABA released by AgRP neurons inhibits kisspeptin neurons^6^; and that NPY knockout or melanocortin receptor depletion (which would reduce AgRP efficacy) both improve fertility in leptin signalling‐deficient mice.^3^, ^25^ Therefore, all three neurochemicals have the potential to act as effectors of AgRP neuronal activity on pubertal timing. During metabolic deficiency, AgRP neurons escalate their negative effect on HPG axis, likely by inhibiting kisspeptin neurons on which they synapse. The lower *Kiss1* expression that has been observed in hypogonadotropic lean animals^38^ as well as leptin‐resistant obese animals^39^ compared to animals of normal bodyweight could be a result of such unrestrained AgRP neuronal activity.

AgRP neurons are known to relay leptin deficiency signals to the reproductive axis, and direct actions of the anorexigenic adipose‐derived hormone leptin on AgRP neurons are sufficient to enable puberty onset and adult fertility in mice of both sexes.^14^ Leptin inhibits the activity of AgRP/NPY neurons, so these neuropeptides cause suppression of the reproductive axis in conditions of leptin deficiency. Consistent with this, ablation of these neurons or knockout of the genes encoding either neuropeptide or the NPY Y4 receptor partially rescues the infertility phenotype of leptin signalling‐deficient mice.^5^, ^13^, ^25^, ^40^

In the present study, we confirmed a delay of puberty onset when undernutrition occurs during neonatal period, which would serve to withhold the capacity to reproduce until energy reserves and perhaps environmental conditions are more favourable. We showed that inhibition of AgRP neuronal activity was able to overcome the adverse effects of nutritional restriction on puberty onset in males. These results suggest an inhibitory role for AgRP neurons, acting as a brake on prepubertal HPG activity at least in male mice.

## AUTHOR CONTRIBUTIONS

**George ADP Connolly:** Formal analysis; investigation; methodology. **Caroline Ancel:** Investigation. **Megan A Inglis:** Investigation; validation. **Greg M Anderson:** Conceptualization; funding acquisition; supervision; writing – review and editing.

## FUNDING INFORMATION

This work was supported by The Royal Society of New Zealand Marsden Fund (17‐UOO‐127).

## CONFLICT OF INTEREST

The authors have nothing to disclose.

### PEER REVIEW

The peer review history for this article is available at https://publons.com/publon/10.1111/jne.13190.

## Data Availability

Some datasets generated during the current study are not publicly available but are available from the corresponding author on reasonable request.
